# The sphingosine-1-phosphate receptor 2 S1PR2 mediates chronic glucocorticoid exposure-induced hepatic steatosis and hypertriglyceridemia

**DOI:** 10.1016/j.jbc.2025.110353

**Published:** 2025-06-07

**Authors:** Maggie Chang, Michelle Zhao, Emily M. Whang, Rebecca A. Lee, Donald K. Scott, Jen-Chywan Wang

**Affiliations:** 1 Endocrinology Graduate Group, University of California Berkeley, Berkeley, California, USA; 2 Department of Nutritional Sciences & Toxicology, University of California Berkeley, Berkeley, California, USA; 3 Diabetes, Obesity and Metabolism Institute, Icahn School of Medicine at Mount Sinai, New York, New York, USA; 4 Metabolic Biology Graduate Group, University of California Berkeley, Berkeley, California, USA

**Keywords:** glucocorticoids, glucocorticoid receptor, Sphingosine-1-phosphate (S1P), liver, Srebp1c, ChREBP, lipid metabolism, hepatic steatosis, dyslipidemia, sphingolipid

## Abstract

Glucocorticoids are potent anti-inflammatory agents that are frequently used to treat inflammatory and autoimmune diseases. Chronic glucocorticoid treatment, however, causes unwanted adverse effects such as hypertriglyceridemia and hepatic steatosis. Here we showed that reducing the expression of sphingosine-1-phosphate receptor 2 (S1PR2) in mice liver reduced chronic glucocorticoid exposure induced triglyceride accumulation in the liver and the plasma. Chronic glucocorticoid treatment increased the recruitment of sterol regulatory element-binding protein 1c (Srebp1c) to the sterol regulatory element of mouse *fatty acid synthase (Fasn)* gene. This response was attenuated in hepatic S1PR2 knockdown mice. Chronic glucocorticoid treatment also increased the recruitment of carbohydrate response element binding protein (ChREBP) to the carbohydrate response elements (ChoREs) of lipogenic and glycolytic genes. This response was partially reduced in hepatic S1PR2 knockdown mice. Reducing hepatic ChREBP expression reduced the expression of *Pklr, Me1,* and *Fasn*. However, long-term glucocorticoid induced triglyceride accumulation in the liver and the plasma were not affected whereas the hepatic lactate levels were decreased. Thus, ChREBP plays a major role in chronic glucocorticoid induced glycolysis whereas its role in hypertriglyceridemia and hepatic steatosis was modest. Overall, this study demonstrated that hepatic S1PR2 signaling plays a partial but significant role in chronic glucocorticoid exposure-activated Srebp1c and ChREBP which promote lipogenesis and glycolysis, respectively.

Glucocorticoids are steroid hormones that play a complex role in the regulation of hepatic lipid metabolism. During short-term glucocorticoid exposure, such as fasting, glucocorticoids inhibit lipogenesis, so the energy can be used for gluconeogenesis (1). In contrast, chronic glucocorticoid exposure, which occurs under long-term pharmacological glucocorticoid therapies against inflammatory and autoimmune diseases, causes hypertriglyceridemia and hepatic steatosis (2, 3, 4, 5). In patients, this limits the application of glucocorticoid pharmacotherapy and can cause comorbidities with other conditions (6, 7, 8). Understanding the mechanisms underlying this long-term glucocorticoid effect on lipid disorders is important for developing improved glucocorticoid therapies.

Glucocorticoid actions are mainly mediated by its intracellular glucocorticoid receptor (GR), which is a transcription regulator. We previously showed that chronic glucocorticoid treatment-induced hepatic steatosis and hypertriglyceridemia are attenuated in mice lacking *angiopoietin-like 4 (Angptl4),* a GR primary target gene that encodes a secreted protein that inhibits lipoprotein lipase and promotes adipose tissue lipolysis (9, 10, 11). Notably, long-term glucocorticoid treatment causes an accumulation of hepatic ceramides (12). These glucocorticoid effects were significantly attenuated in *Angptl4* null mice (12). Serine palmitoyltransferase (SPT), the rate-controlling enzyme of ceramide biosynthesis, initiates sphingoid base formation from palmitoyl-CoA and serine. This forms 3-keto-dihydrosphingosine which is further converted by several enzymes to form ceramides. These substrates are increased by prolonged glucocorticoid treatment from excess lipolysis of white adipose tissue and muscle proteolysis (13, 14). Inhibiting ceramide synthesis using a pharmacological inhibitor of SPT or genetic knockdown of *Spt long chain subunit 2* (*Sptlc2*) alleviates chronic glucocorticoid-induced hepatic steatosis and hypertriglyceridemia (12). The downstream effector of ceramides, protein kinase C ζ (PKCζ), is involved in glucocorticoid-induced plasma and liver triglyceride (TG) accumulation (15). PKCζ has been shown to phosphorylate Baf60c, a component in SWI/SNF transcriptional coregulator complex, to increase its nuclear localization (16). Baf60c interacts and coactivates the transcription factor upstream stimulatory factor 1 and 2 (USF1/2) to stimulate the transcription of lipogenic genes, such as *fatty acid synthase (Fasn)* (12, 16).

Ceramides can be further converted to sphingosines, which are phosphorylated to sphingosine-1-phosphate (S1P) by sphingosine kinase 1 and 2 (Sphk1/2). S1P can exert its actions intracellularly and extracellularly (17, 18). In the extracellular space, S1P binds to its cognate membrane receptors, S1P receptors (S1PRs), which are G protein-coupled receptors (18, 19). There are five S1PRs, S1PR1-5 (20). Previously, we have established the role of S1PR2 signaling in enhancing hepatic gluconeogenesis during chronic glucocorticoid exposure (21). During these studies, we found that long-term dexamethasone (Dex, a synthetic glucocorticoid) treatment increased the expression of many lipogenic genes, which were reduced in hepatic S1PR2 knockdown mice (21). These results prompt us to further analyze the role of S1PR2 in chronic glucocorticoid-induced hepatic steatosis and hypertriglyceridemia.

Here, we found that hepatic knockdown of S1PR2 indeed alleviated long-term Dex treatment-induced plasma and liver TG accumulation. We analyzed the effect of hepatic S1PR2 knockdown on the activation of sterol regulatory element-binding protein 1c (Srebp1c) by long-term Dex treatment. Interestingly, RNA sequencing (RNA-seq) found that in addition to lipogenic and gluconeogenic genes, chronic Dex treatment also increased the expression of genes encoding enzymes in glycolysis and fructose metabolism, which are target genes of transcription factor carbohydrate-responsive element binding protein (ChREBP) in mouse livers. In hepatic S1PR2 knockdown mice, the ability of Dex to induce some of these genes was compromised in the liver. Thus, the role of ChREBP in chronic glucocorticoid exposure-induced lipid disorders was further explored.

## Results

### Hepatic S1PR2 knockdown reduced chronic Dex-induced plasma and liver triglycerides

**Figure 1:**
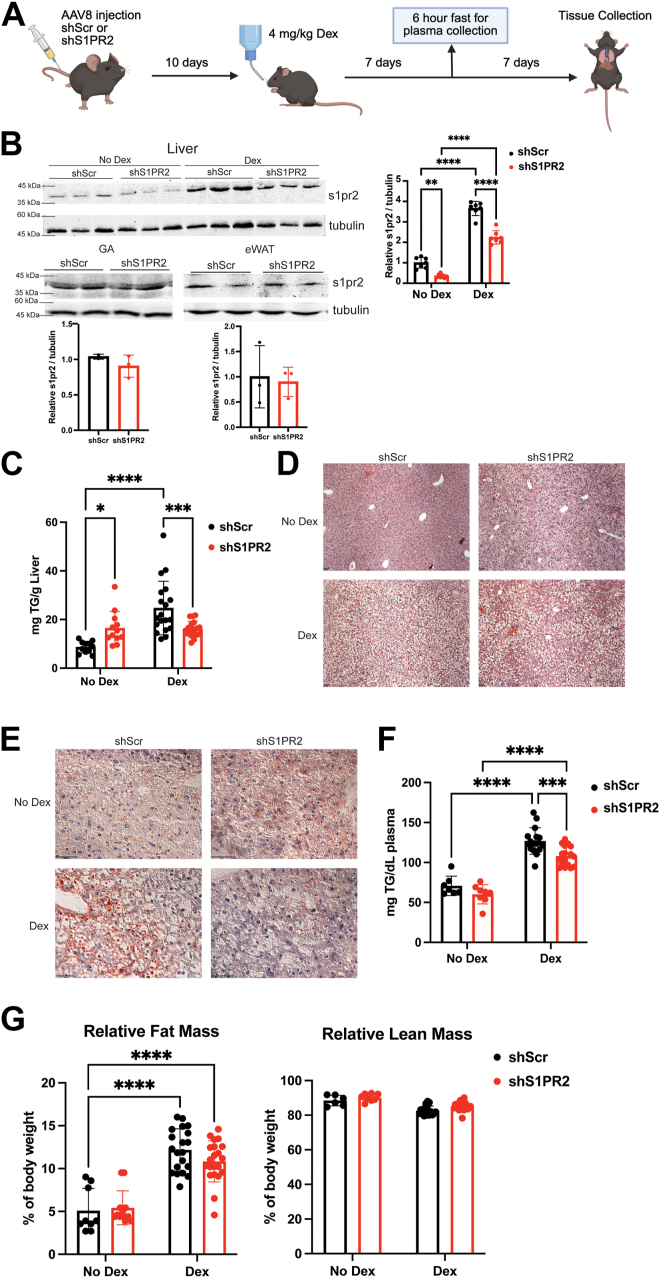
**Hepatic S1PR2 knockdown reduces chronic Dex-induced triglyceride accumulation.***A*, Male WT mice were injected with AAV8 and treated with or without 4 mg/kg Dexamethasone *via* drinking water for 2 weeks. *B*, representative western blots in liver, gastrocnemius muscle, and epidydimal white adipose tissue of shScr and shS1PR2 mice and band density quantification of liver blot using ImageJ, n = 6 to 7. *C*, liver triglyceride levels, n = 11 to 19. Representative liver staining with (*D*) H&E (magnification 10x, scale bar 50 μM) and (*E*) Oil Red O (magnification 40x, scale bar 50 μM). *F*, fasted plasma triglyceride levels, n = 7 to 19. *G*, echo MRI measurements of relative fat and relative lean mass, n = 9 to 21. ∗*p* < 0.05, ∗∗*p* < 0.01, ∗∗∗*p* < 0.001, ∗∗∗∗*p* < 0.0001 by two-way ANOVA with Fisher’s LSD test. Data presented as mean with S.D.

Wild-type (WT) male mice were injected with adeno-associated virus serotype 8 (AAV8) expressing scramble small hairpin RNA (shRNA, shScr, control) or shRNA targeting S1PR2 (shS1PR2). 10 days post-injection, mice were treated with or without dexamethasone (Dex, a synthetic glucocorticoid) for 2 weeks (Fig. 1*A*). Western blot shows the reduction of S1PR2 protein levels in the liver, but not gastrocnemius muscle or epidydimal white adipose tissue (Fig. 1*B*). Thus, S1PR2 knockdown is specific to the liver. Chronic Dex treatment caused a significant increase of TG levels in the livers of shScr mice while hepatic S1PR2 knockdown reduced this effect (Fig. 1*C*). H&E staining of livers showed an increase in vacuole size in Dex treated shScr mice, which indicates increased neutral lipid accumulation. This was attenuated in Dex-treated shS1PR2 mice (Fig. 1*D*). Similarly, Oil Red O staining showed increased levels of lipids in the livers of Dex-treated shScr mice but reduced lipid levels in livers of Dex treated shS1PR2 mice (Fig. 1*E*). Interestingly, hepatic S1PR2 knockdown appears to increase liver TG levels. These results were similar to the observation in *S1PR2* null mice (22). One week of Dex treatment also increased fasting plasma TG levels (Fig. 1*F*). This induction was reduced by hepatic S1PR2 knockdown (Fig. 1*F*). Thus, reducing hepatic S1PR2 levels is protective of Dex-induced plasma TG accumulation. Echo MRI analysis showed that Dex treatment increased relative fat mass but hepatic S1PR2 knockdown did not affect fat mass in control or Dex treated mice (Fig. 1*G*). Overall, these results demonstrate that hepatic knockdown of S1PR2 attenuated chronic Dex treatment induction of TG levels in the liver and the plasma.

### Hepatic S1PR2 knockdown reduced Dex-induced Srebp1c activation

**Figure 2:**
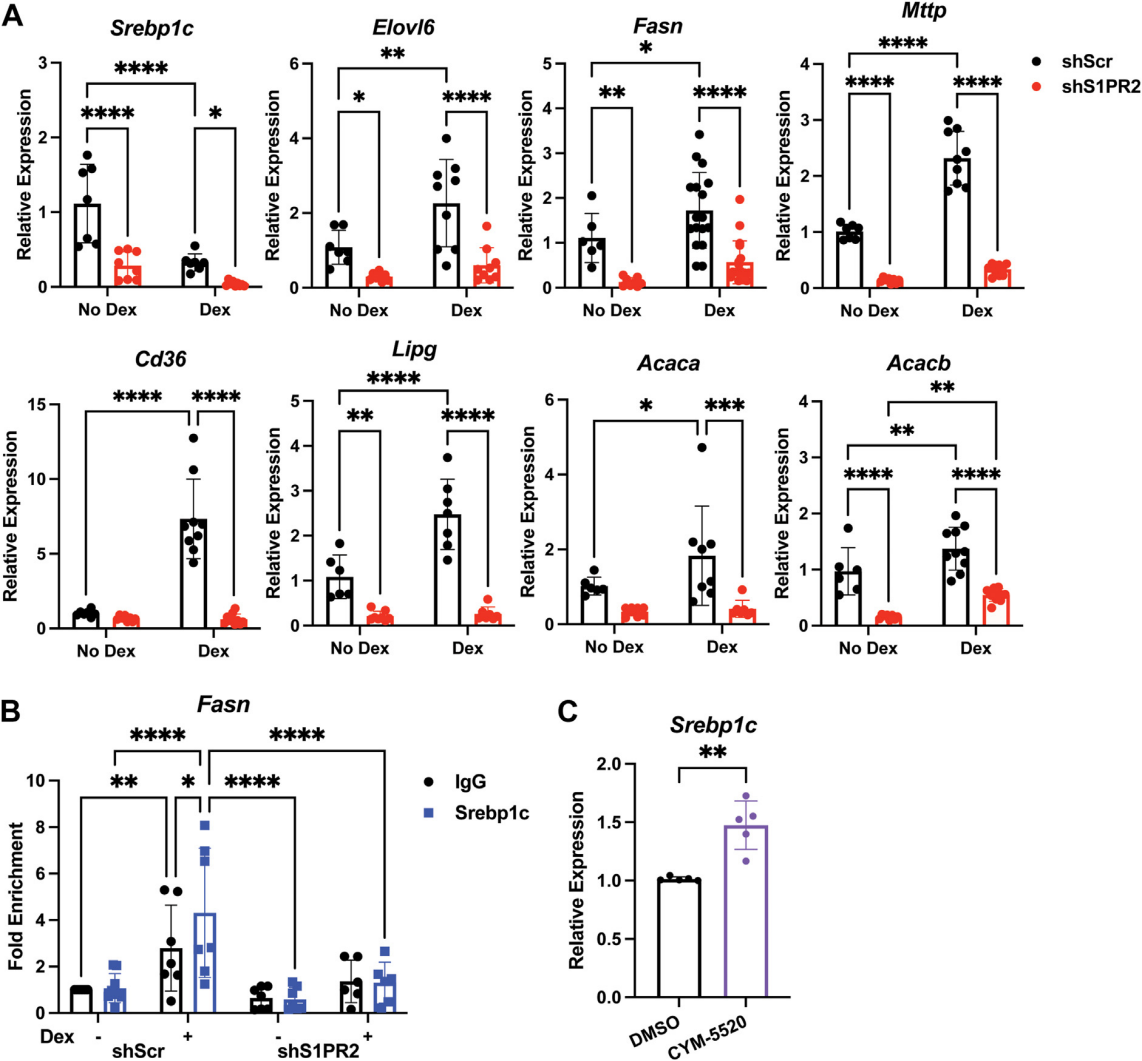
**Hepatic S1PR2 knockdown reduces chronic Dex-induced expression of lipid metabolism genes.***A*, mRNA levels in livers of shScr and shS1PR2 mice treated with or without Dex, n = 7 to 18. *B*, ChIP for Srebp1c in livers of shScr and shS1PR2 mice treated with or without Dex, n = 7. ∗*p* < 0.05, ∗∗*p* < 0.01, ∗∗∗∗*p* < 0.0001 using two-way ANOVA with Fisher’s LSD test. (C) mRNA levels in mouse primary hepatocytes treated with or without CYM-5520, n = 5, ∗∗*p* < 0.001 using unpaired *t* test with Welch’s correction. Data presented as mean with S.D.

The expression of genes involved in lipid metabolism such as *Srebp1c, Elovl6, Fasn, Mttp, Gpat1, Cd36, Lipg, Acaca,* and *Acacb* were analyzed in the livers of Dex-treated shScr and shS1PR2 mice. We found that Dex treatment increased their expression (Fig. 2*A*). These results agreed with our previous observation (15). S1PR2 knockdown suppressed these Dex effects (Fig. 2*A*). Interestingly, hepatic S1PR2 knockdown also suppressed these genes without Dex treatment (Fig. 2*A*). Our previous studies showed that Srebp1c activity was induced by chronic Dex treatment, despite its reduction in mRNA expression by Dex (15). Indeed, we found that Srebp1c was recruited to the sterol response element (SRE) of the mouse *Fasn* gene in the liver under 2 weeks Dex treatment (Fig. 2*B*). This induction was attenuated in hepatic S1PR2 knockdown mice (Fig. 2*B*). Thus, S1PR2 signaling is involved in chronic Dex-induced Srebp1c activity.

We tested whether activating S1PR2 signaling affects the expression of Srebp1c independent of Dex treatment. Mouse primary hepatocytes were treated with or without CYM-5520, a S1PR2 agonist, for 4 h (23). After RNA was isolated from these cells, real-time PCR was performed to monitor *Srebp1c* expression. We found that Srebp1c expression was significantly elevated by CYM-5520 treatment (Fig. 2*C*).

### Sphk2 is not involved in chronic Dex-induced lipid disorders

**Figure 3:**
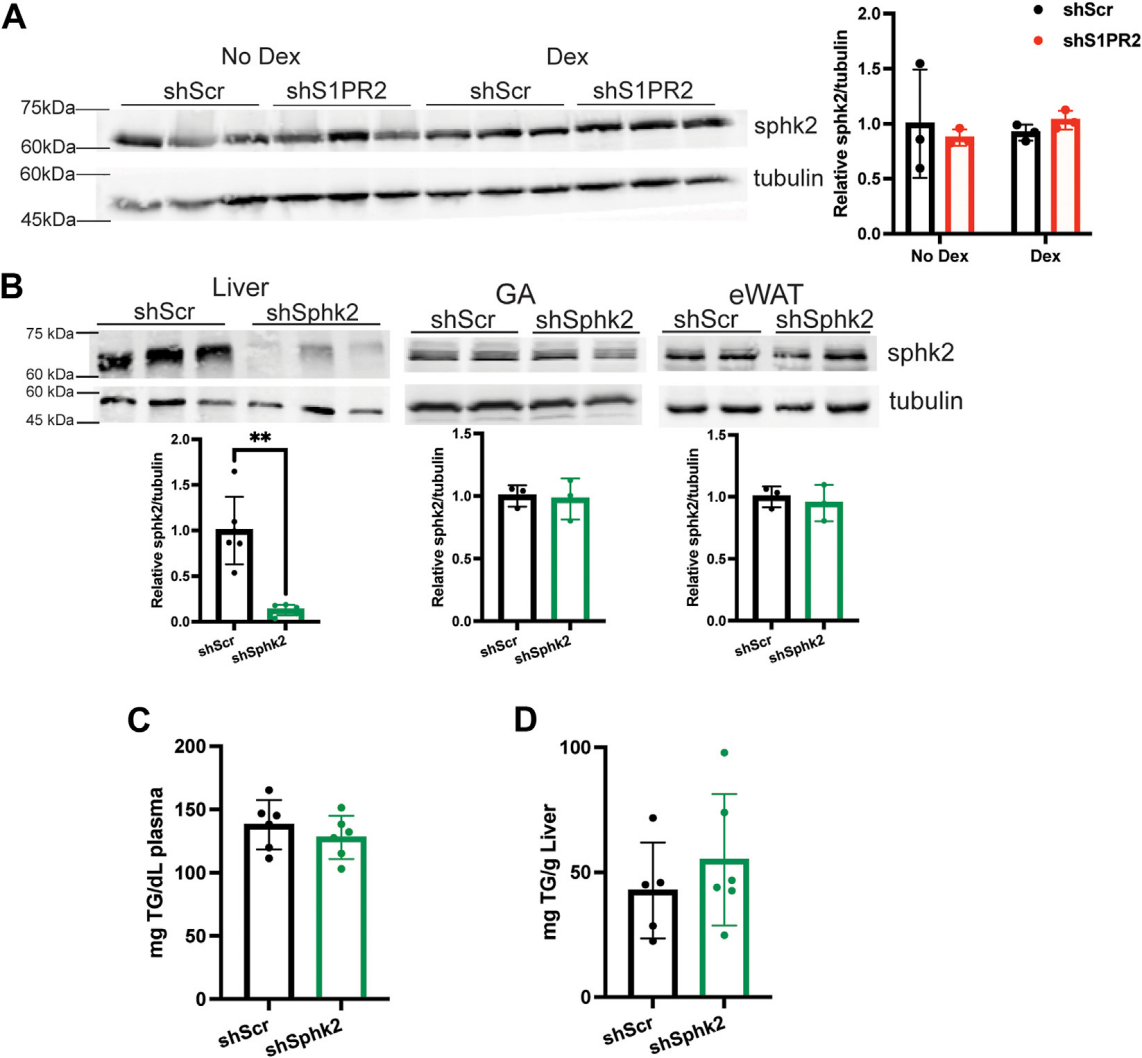
**Hepatic Sphk2 is not involved in chronic Dex-induced triglyceride accumulation.***A*, western blot in livers of shScr and shS1PR2 mice treated with or without Dex and band density quantification using ImageJ, n = 3 using two-way ANOVA with Fisher’s LSD test. *B*, representative western blots in the liver, gastrocnemius muscle, and epidydimal white adipose tissue of Dex-treated shScr and shSphk2 mice and band density quantification using ImageJ, n = 3 to 5. *C*, fasted plasma triglyceride levels, n = 6 (*D*) liver triglyceride levels, n = 6. ∗∗*p* < 0.01 using unpaired *t* test with Welch’s correction. Data presented as mean with S.D.

Previous studies have shown that sphingosine kinase 2 (Sphk2) is required for conjugated bile acids-activated S1PR2 signaling to stimulate lipogenesis in hepatocytes (22, 24). Western blotting showed no changes to hepatic Sphk2 levels by chronic Dex treatment or hepatic S1PR2 knockdown (Fig. 3*A*). Next, we determined whether Sphk2 is involved in chronic Dex treatment-induced TG accumulation by injecting WT mice with AAV8 expressing scramble shRNA (shScr, control) or shRNA that targeted Sphk2 (shSphk2) through the tail vein. 10 days post injection, shScr and shSphk2 mice were placed on Dex for 2 weeks. Sphk2 protein levels were reduced in the liver, but not in gastrocnemius muscle or epididymal white adipose tissue (Fig. 3*B*). There was no difference in plasma and liver TG levels between Dex-treated shScr and Dex-treated shSphk2 mice (Fig. 3, *C* and *D*). Thus, Sphk2 does not play a role in chronic Dex-induced lipid disorders and S1PR2’s role in mediating chronic Dex-induced hypertriglyceridemia and hepatic steatosis likely does not require Sphk2.

### S1PR2 is involved in chronic Dex activation of ChREBP

**Figure 4:**
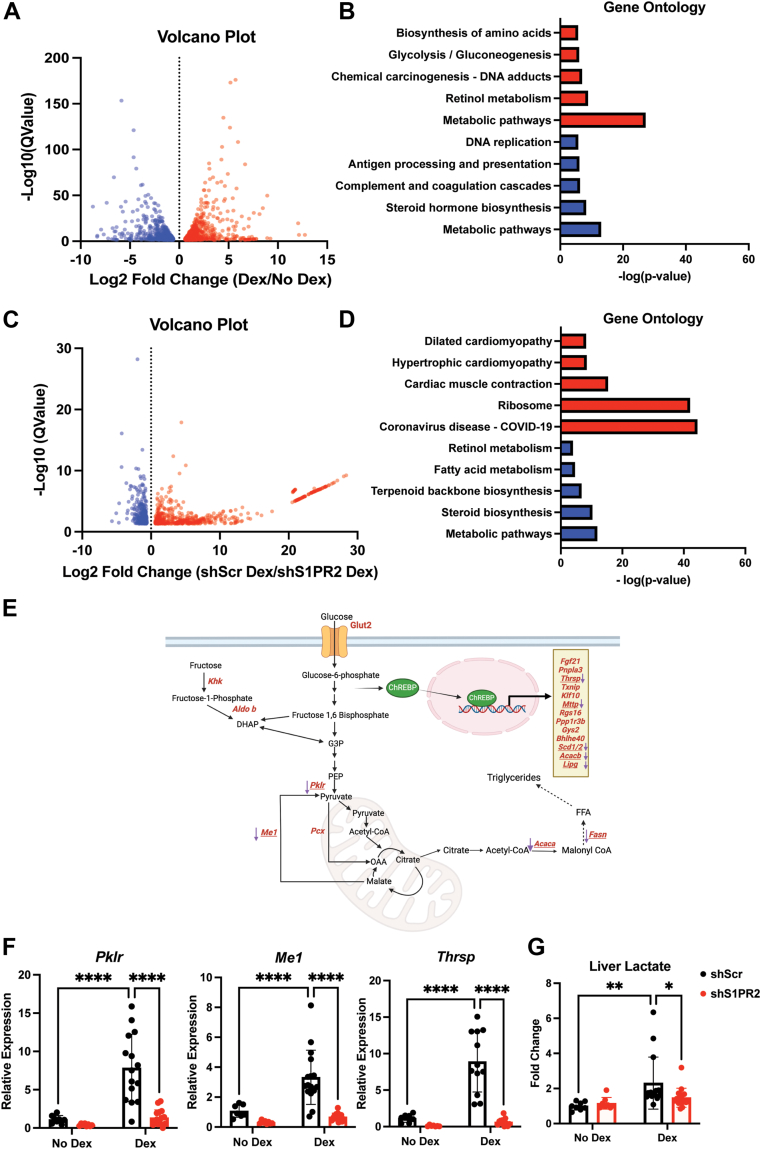
**RNA sequencing identifies ChREBP target genes to be activated by Dex and reduced by hepatic S1PR2 knockdown**. *A*, volcano plot of differentially expressed genes in livers of shScr mice treated with or without Dex with 1.5-fold difference and adjusted *p*-value of 0.05 as a cutoff. *B*, gene ontology analysis by Kegg Pathways of genes induced or reduced between shScr mice treated with or without Dex. *C*, volcano plot of differentially expressed genes in livers of shScr and shS1PR2 Dex-treated mice with 1.5-fold difference and adjusted *p*-value of 0.05 as a cutoff. *D*, gene ontology analysis by Kegg Pathways of genes induced and reduced between livers of shScr and shS1PR2 Dex-treated mice. *E*, ChREBP target differentially expressed genes involved in glycolysis and fructolysis that are regulated by chronic Dex treatment and hepatic S1PR2 knockdown. Genes in *red font* are upregulated by chronic Dex treatment. *Purple arrows* and *underlining* indicate genes that are downregulated by hepatic S1PR2 knockdown. Figure created by BioRender. *F*, mRNA levels in shScr and shS1PR2 livers treated with or without Dex, n = 8 to 16. *G*, Relative lactate levels in livers of shScr and shS1PR2 mice treated with or without Dex, n = 8 to 16. ∗*p* < 0.05, ∗∗*p* < 0.01, ∗∗∗∗*p* < 0.0001 by two-way ANOVA with Fisher’s LSD test. Data presented as mean with S.D.

To explore whether S1PR2 is involved in other pathways that are regulated by chronic Dex-treatment, we performed RNA sequencing (RNA-seq) to identify differentially expressed genes in the livers of control and Dex-treated shScr and shS1PR2 mice. Based on an adjusted *p* value and 1.5-fold cut off, we found that 951 genes were increased whereas 780 genes were reduced by Dex treatment (Fig. 4*A*). Gene ontology analysis showed that glycolytic/gluconeogenic genes and genes involved in retinol and amino acid biosynthesis were increased by Dex treatment (Fig. 4, *B* and *E*). Moreover, the genes encoding enzymes in lipogenesis, such as *Fasn*, *Acaca*, and *Me1*, were also increased, which agrees with the results shown above. For genes reduced by Dex, those involved in steroid hormone biosynthesis and complement and coagulation cascades were highly represented (Fig. 4*B*). We were very interested in the induction of glycolytic and fructose metabolism genes by long-term Dex treatment. These genes are targets of carbohydrate response element binding protein (ChREBP), which can also activate lipogenic genes (25, 26). There were at least 23 known ChREBP target genes that were induced by Dex treatment (Fig. 4*E*). A full list of the differentially regulated genes is available in File S1.

We next compared the gene expression profiles in the livers of Dex-treated shScr and Dex-treated shS1PR2 mice. We found that 594 genes were upregulated whereas 340 genes were downregulated in the liver of Dex-treated shS1PR2 mice (Fig. 4*C*). Gene ontology analysis identified the top five most upregulated and downregulated biological processes. Upregulated biological processes in order of significance were cytoplasmic translation, translation, muscle contraction, sarcomere organization and ribosomal small subunit biogenesis (Fig. 4*D*). Reducing hepatic S1PR2 expression significantly attenuated the ability of chronic Dex exposure to promote the expression of genes involved in triglyceride, cholesterol, and sterol biosynthesis (Fig. 4, *D* and *E*). Interestingly, a subset of ChREBP target genes induced by chronic Dex exposure was reduced by hepatic S1PR2 knockdown (Fig. 4*E*).

We performed RT-PCR to confirm downregulated genes by hepatic S1PR2 knockdown identified by RNA-seq. Indeed, the expression of several ChREBP target genes, such as *Me1, Pklr,* and *Thrsp*, were reduced by S1PR2 knockdown (Fig. 4*F*). As glycolytic genes, such as *Pklr*, were induced by Dex and reduced by S1PR2 knockdown, we monitored the levels of lactate in the livers of shScr and shS1PR2 mice treated with or without Dex. Inducing glycolysis will increase lactate production. Indeed, Dex treatment increased lactate levels in the liver in shScr mice, and this response was attenuated in shS1PR2 mice (Fig. 4*G*). These results demonstrated that chronic Dex treatment increased glycolysis, which was reduced by S1PR2 knockdown.

**Figure 5:**
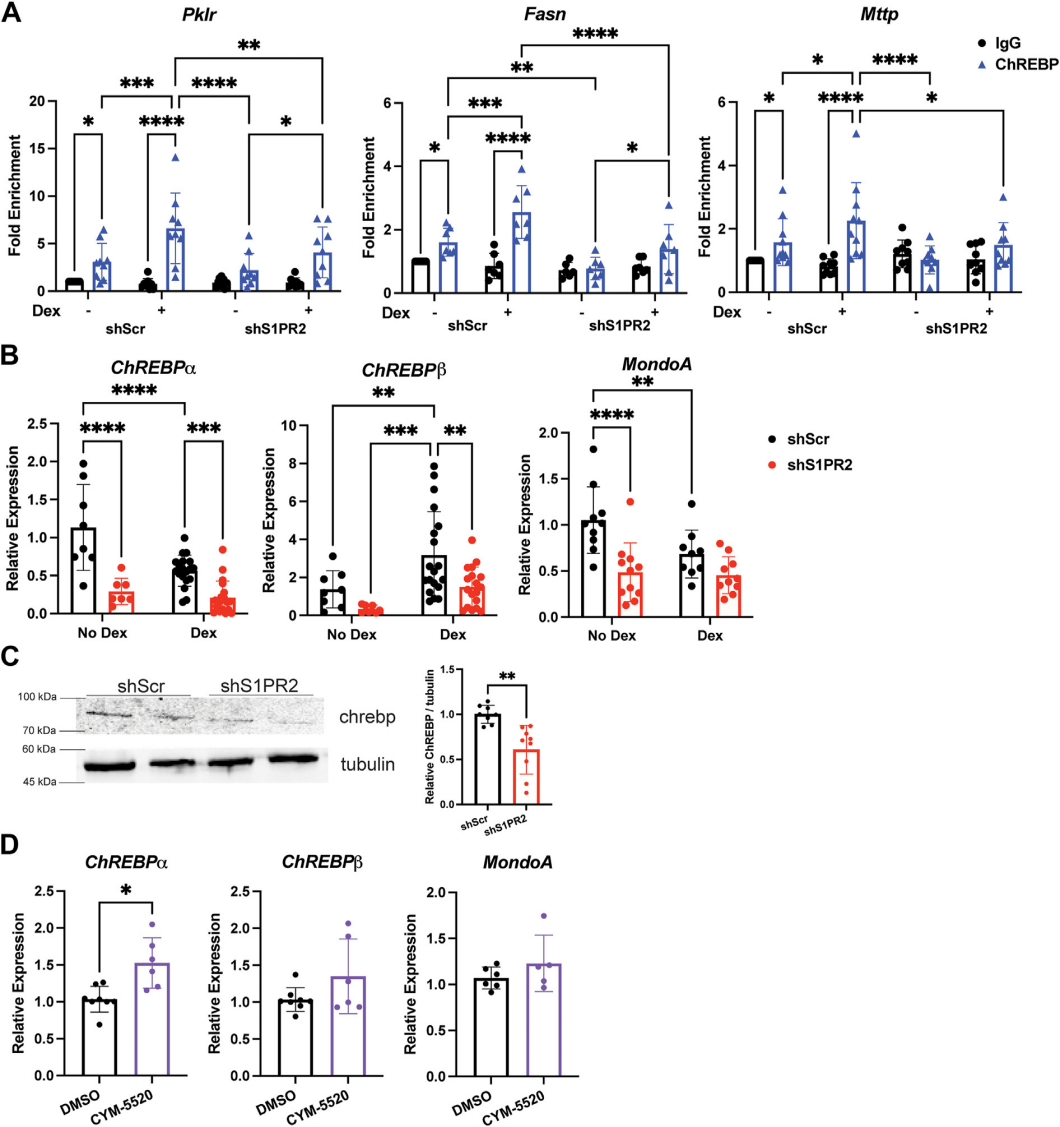
**Dex-induced ChREBP recruitment to target genes is reduced by hepatic S1PR2 knockdown.***A*, ChIP of ChREBP to ChoREs in livers of shScr and shS1PR2 mice treated with or without Dex, n = 7 to 10. *B*, hepatic mRNA levels in shScr and shS1PR2 mice treated with or without Dex, n = 8 to 19. ∗*p* < 0.05, ∗∗*p* < 0.01, ∗∗∗*p* < 0.001, ∗∗∗∗*p* < 0.0001 by two-way ANOVA with Fisher’s LSD test. *C*, representative western blots in liver of Dex-treated shScr and shS1PR2 mice and band density quantification by ImageJ, n = 9. *D*, mRNA levels in mouse primary hepatocytes treated with or without CYM-5520, n = 6. ∗*p* < 0.05 by unpaired *t* test with Welch’s correction. Data presented as mean with S.D.

We performed liver chromatin immunoprecipitation to examine the activation of ChREBP by chronic Dex exposure. Without Dex treatment, ChREBP was present on the promoter of *Pklr, Fasn,* and *Mttp* (Fig. 5*A*). ChREBP is activated by metabolites such as glucose-6-phosphate and xylulose 5-phosphate (25, 26, 27). Therefore, the presence of basal levels of ChREBP on the carbohydrate response-binding element (ChoRE) of these genes was not surprising. We found that ChREBP had significantly higher recruitment to the promoters of these target genes in response to chronic Dex treatment in shScr liver and this Dex-induced occupancy was reduced in livers of Dex-treated shS1PR2 mice (Fig. 5*A*). Thus, ChREBP is activated by long-term Dex treatment and this induction requires S1PR2.

ChREBP contains two isoforms: ChREBPα and ChREBPβ. ChREBPβ is the more transcriptionally powerful isoform of ChREBPα and contains a ChoRE on its promoter (28). RNA-seq did not identify changes in the expression of ChREBPα and ChREBPβ. Nonetheless, we performed conventional real-time PCR analysis to monitor the expression of *ChREBPα* and *ChREBP*β in the liver of control and Dex-treated shScr and shS1PR2 mice. Interestingly, chronic Dex treatment suppressed ChREBPα but increased ChREBPβ expression in the liver of shScr mice while hepatic S1PR2 knockdown reduced *ChREBP*β expression and even further reduced *ChREBP*α expression in the livers of Dex-treated mice (Fig. 5*B*). Protein levels of ChREBP were also reduced in Dex-treated shS1PR2 mice (Fig. 5*C*). Thus, S1PR2 signaling was required to maintain *ChREBP*α and *ChREBP*β expression and the reduction of their expressions in shS1PR2 mice contributes to the reduced recruitment of ChREBP to certain ChoREs during long-term Dex treatment. Because ChREBP expression was reduced, we also analyzed the expression of MondoA, the paralog of ChREBP that also heterodimerizes on the promoter with their same binding partner, MLX (29). While *MondoA* expression was reduced by hepatic S1PR2 knockdown in the basal condition, there was no difference in expression between the livers of shScr and shS1PR2 Dex-treated mice (Fig. 5*B*).

To determine whether activation of S1PR2 signaling affects the expression of ChREBP and MondoA, we isolated mouse primary hepatocytes and treated them with or without the S1PR2 agonist, CYM-5520, for 4 h. Real-time PCR analysis showed a modest, but significant, increase in expression of ChREBPα but not of ChREBPβ or MondoA (Fig. 5*D*).

### Hepatic ChREBP knockdown affected Dex-induced glycolysis but not accumulation of TG

**Figure 6:**
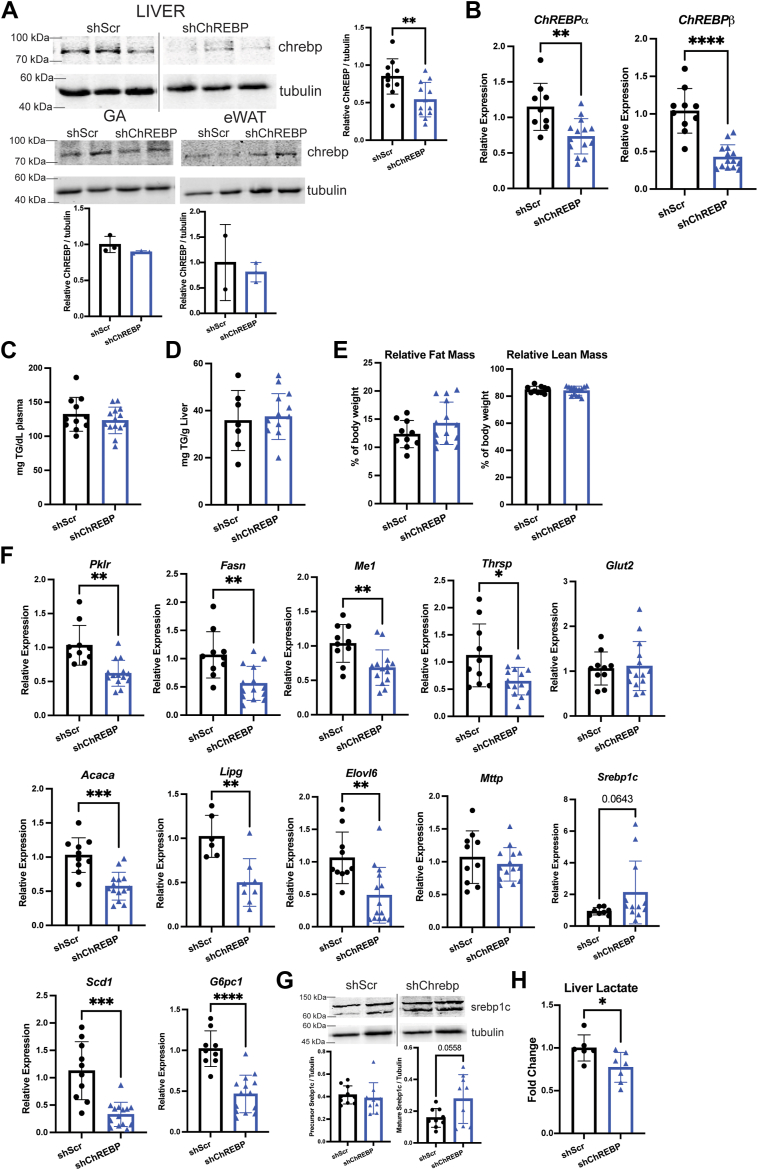
**Hepatic ChREBP knockdown affects chronic Dex-induced glycolysis, but not triglyceride accumulation**. *A*, representative Western blots in the liver, gastrocnemius muscle, and epidydimal white adipose tissue of Dex-treated shScr and shChREBP mice and band density quantification by ImageJ, n = 2 to 12. *B*, mRNA levels in livers of shScr and shChREBP mice, n = 9 to 14. *C*, fasted plasma triglyceride levels, n = 11 to 14. *D*, liver triglyceride levels, n = 7 to 12. *E*, echo MRI measurements of relative fat and relative lean mass, n = 10 to 14. *F*, mRNA levels in livers of shScr and shChREBP mice treated with Dex, n = 10 to 17. *G*, representative western blots in livers of shScr and shChREBP mice treated with Dex and band density quantification by ImageJ, n = 9. *H*, relative lactate levels in livers of shScr and shChREBP mice treated with Dex, n = 6. ∗*p* < 0.05, ∗∗*p* < 0.01, ∗∗∗*p* < 0.001, ∗∗∗∗*p* < 0.0001 using unpaired *t* test with Welch’s correction. Data presented as mean with S.D.

To determine whether ChREBP mediates chronic glucocorticoid exposure-induced lipid disorders, we injected WT male mice with AAV8 expressing shRNA that targeted scramble sequence (shScr) for control or exon eight of ChREBP (shChREBP) which targets both isoforms. 10 days post-injection, the mice were treated with Dex for 2 weeks. Western blot showed reduction of ChREBP expression in liver and not gastrocnemius muscle or epidydimal white adipose tissue (Fig. 6*A*). Notably, real-time PCR showed reduction of both ChREBPα and ChREBPβ in livers of shChREBP mice (Fig. 6*B*). Hepatic ChREBP knockdown in Dex-treated mice had no effect on fasted plasma and liver TG levels (Fig. 6, *C* and *D*). Echo MRI analysis showed no differences in fat or lean mass (Fig. 6*E*). Expression of ChREBP target genes involved in glycolysis and lipogenesis such as *Fasn*, *Pklr*, *Thrsp* and *Me1* that were induced by chronic Dex treatment were reduced by hepatic ChREBP knockdown (Fig. 6*F*). Interestingly, expression of *Srebp1c* was trending to be higher in livers of shChREBP mice (Fig. 6*F*). Likewise, protein levels of the cleaved and mature form of Srebp1c were trending to be higher in livers of shChREBP mice (Fig. 6*G*). Hepatic lactate levels were reduced in liver of shChREBP mice (Fig. 6*H*). Thus, while knockdown of ChREBP reduces Dex-induced glycolysis, it does not attenuate chronic Dex-induced hypertriglyceridemia and hepatic steatosis despite the decreased expression of certain lipogenic genes.

**Figure 7:**
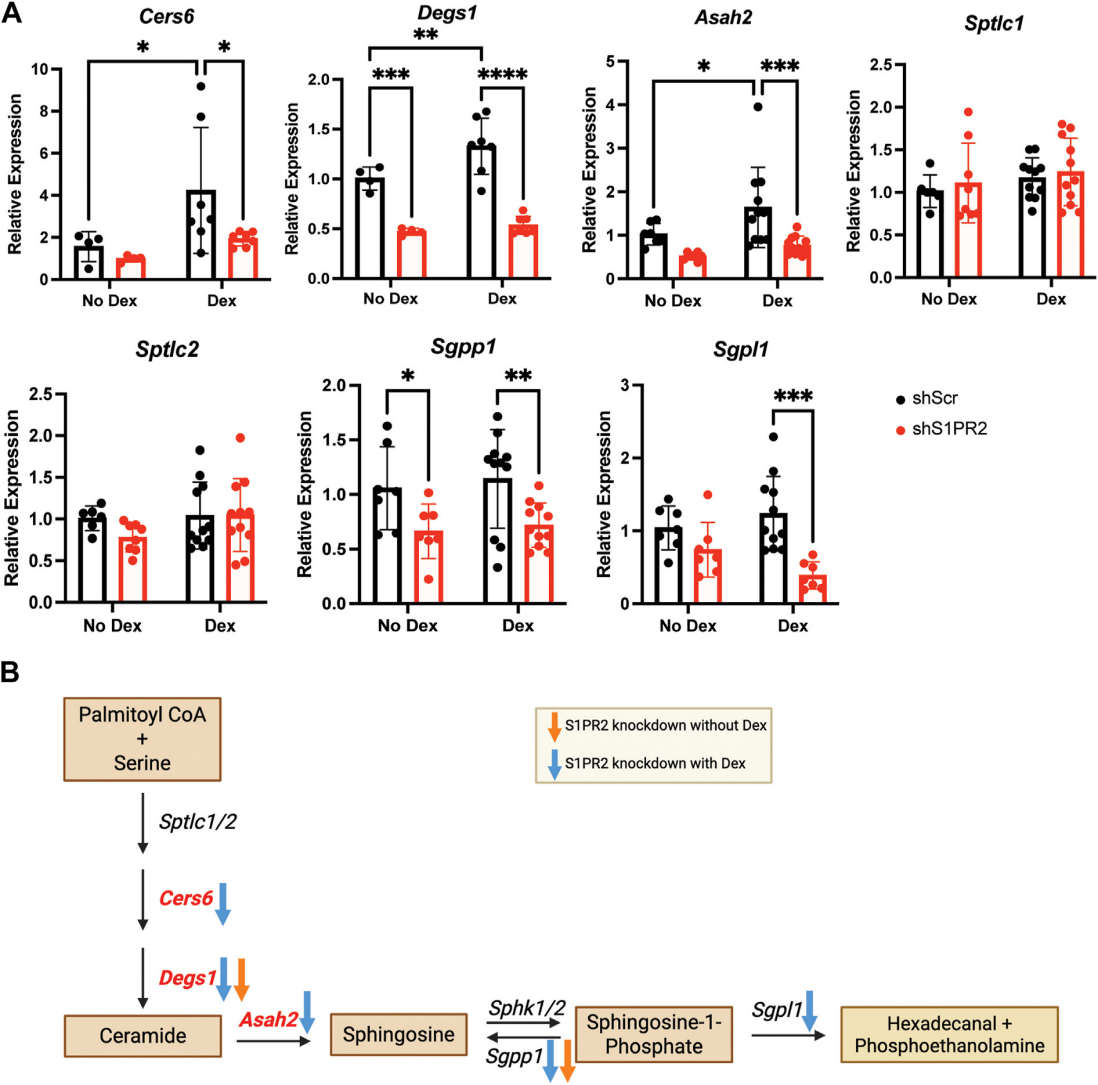
**Chronic Dex treatment and hepatic S1PR2 knockdown effects on sphingolipid metabolic enzyme gene expression**. *A*, mRNA levels of sphingolipid metabolic enzymes in livers of shScr and shS1PR2 mice treated with or without Dex, n = 4 to 11. *B*, effects of hepatic S1PR2 knockdown on the sphingolipid metabolism pathway. Dex-induced genes are in *bold* and *red font*. *Orange arrow* indicates genes reduced by S1PR2 knockdown without Dex treatment. *Blue arrow* indicates genes reduced by S1PR2 knockdown with Dex treatment. Figure created using BioRender. ∗*p* < 0.05, ∗∗*p* < 0.01, ∗∗∗*p* < 0.001, ∗∗∗∗*p* < 0.0001 using two way ANOVA with Fisher’s LSD test. Data presented as mean with S.D.

### Hepatic S1PR2 knockdown suppressed Dex-induced sphingolipid biosynthetic genes

We performed gene expression analysis to monitor the effect of hepatic S1PR2 knockdown in mice treated with or without Dex. For mice that were not treated with Dex, S1PR2 knockdown reduced the expression of *Delta 4-desaturase sphingolipid 1* (*Degs1*) and *sphingosine-1-phosphate phosphatase 1* (*Sgpp1*). Dex treatment increased the expression of *ceramide synthase 6* (*Cers6*), *Degs1,* and *N-acylsphingosine amidohydrolase 2* (*Asah2,* a.k.a. neutral ceramidase) was significantly induced by chronic Dex treatment (Fig. 7*A*). Hepatic S1PR2 knockdown reduced the Dex induction of these genes. Hepatic S1PR2 knockdown significantly reduced the expressions of the S1P degradation enzymes *sphingosine-1-phosphate lyase 1* (*Sgpl1*) and *Sgpp1* though Dex treatment did not affect their expression (Fig. 7*A*). Overall, based on the gene expression analysis, S1PR2 knockdown likely decrease ceramides and S1P production in the liver (Fig. 7*B*). Chronic Dex treatment increased ceramides and S1P production which is mostly consistent with our previous observation (12). Hepatic S1PR2 knockdown likely antagonized these Dex effects. Metabolomics analysis is needed to confirm these predications in future studies.

**Figure 8:**
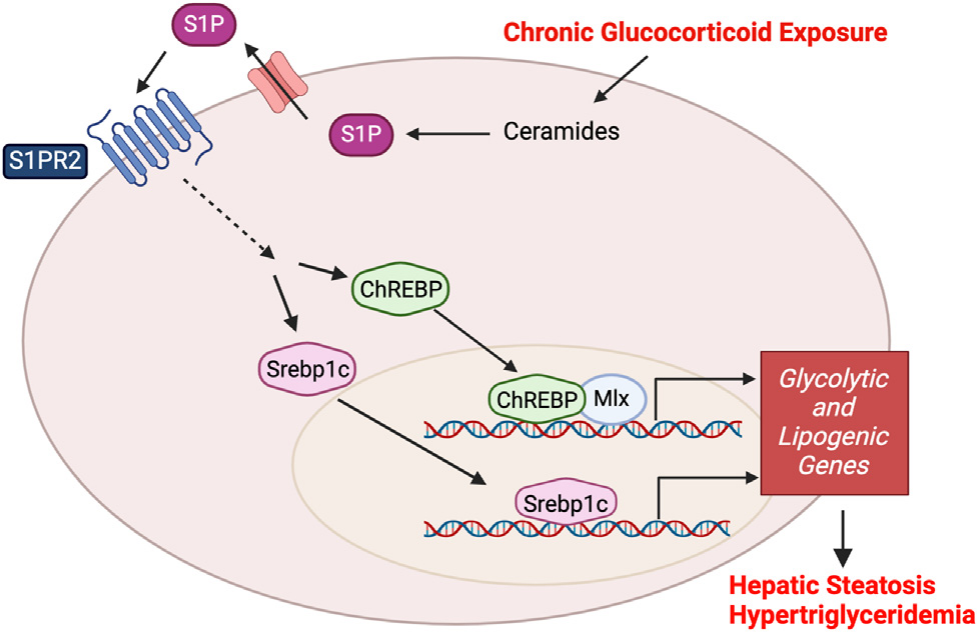
**Model of chronic Dex exposure-induced S1PR2 signaling on hepatic steatosis and hypertriglyceridemia.** Chronic Dex exposure induces S1PR2 signaling which activates ChREBP and Srebp1c to bind to their target promoter genes. These genes are involved in glycolysis and lipogenesis that contribute toward the lipid disorders of hepatic steatosis and hypertriglyceridemia. Figure created using BioRender.

In summary, these results demonstrate that chronic Dex treatment activates S1PR2 signaling which increases the occupancy of ChREBP and Srebp1c to the promoters of target lipogenic and glycolytic genes. This increase in gene expression contributes toward the development of chronic Dex exposure-induced lipid disorders (Fig. 8).

## Discussion

In this study, we established the role of hepatic S1PR2 signaling in chronic glucocorticoid exposure-induced hypertriglyceridemia and hepatic steatosis. Previous studies have shown that conjugated bile acids bind to S1PR2 to activate extracellular signal-regulated kinase half (ERK1/2) which phosphorylates Sphk2 to increase its nuclear localization (22). In the nucleus, Sphk2 converts sphingosine to S1P, which binds to and inhibits HDAC1/2 to activate lipogenic gene transcription (22). However, our experiments demonstrated that Sphk2 is not require for chronic Dex treatment-induced TG accumulation in the liver and the plasma but rather a different mechanism is induced. Our experiments did find that chronic Dex treatment activated Srebp1c, which agrees with our previous report, and that S1PR2 is required for this activation (15). We found that treatment with a S1PR2 agonist increased the mRNA levels of Srebp1c in mouse primary hepatocytes, which aligns with the previous study that showed Srebp1c expression is elevated by S1PR2 overexpression in mouse primary hepatocytes (22). Notably, although Sphk2 was not involved in chronic Dex-induced hepatic steatosis and hypertriglyceridemia, we cannot exclude the involvement of ERK1/2 and HDAC1/2 in this process. The previous report proposed that the role of Sphk2 was to generate S1P in the nucleus. Perhaps, in chronic Dex treatment, there are enough S1P levels in the nucleus and therefore Sphk2 activation is not needed. However, HDAC1/2 are still required to increase Srebp1c expression. In the meantime, ERK1/2 could initiate Sphk2 independent pathways to increase Srebp1c. Notably, S1PR2 signaling activates 3 Gα subunits: G_i_, G_12/13_, and G_q_ (30, 31). G_i_ activates MAP kinase ERK1/2 whereas G_12/13_ activates GTPase Rho (30, 31). G_q_ promotes the activity of phospholipase C (PLC) (30, 31). In any case, more studies are needed to determine the downstream effectors of S1PR2 that increase Srebp1c expression under chronic Dex treatment. Thus, in addition to the ceramide-PKCζ axis, S1PR2 signaling is another mechanism by which long-term Dex treatment activates Srebp1c.

Interestingly, without Dex, hepatic S1PR2 knockdown suppressed basal lipogenic gene expression but had increased liver TG accumulation. This observation aligns with a previous report of whole-body S1PR2 knockout mice developing hepatic steatosis (22). It is unclear the mechanism underlying this phenotype. Based on RNA-seq data, in addition to lipogenic genes, a list of the gene involved in fatty acid oxidation and mitochondrial oxidative phosphorylation was also reduced by S1PR2 knockdown, such as *NADH:Ubiquinone oxidoreductase subunit b1(Ndufb1), peroxisome proliferator-activated receptor α (Ppara), electron transfer flavoprotein dehydrogenase (Etfdh), acyl-CoA oxidase 1 (Acox1)* and *very long-chain acyl-CoA dehydrogenase (Acadvl)* (File S1, sheet “shScr *versus* shS1PR2 No Dex”). Reduction of fatty acid oxidation and oxidative phosphorylation could potentially result in the accumulation of TG in the liver. This notion, however, needs to be confirmed in future studies. Notably, the expression of most of these fatty acid oxidation and oxidative phosphorylation genes in the liver of Dex-treated mice was not affected by S1PR2 knockdown.

One novel finding in this study is that chronic Dex treatment promotes the expression of genes involved in glycolysis and fructose metabolism. Many of these genes are ChREBP target genes. Notably, the activation of ChREBP by chronic glucocorticoid treatment has not been previously reported. ChIP confirmed that ChREBP is activated by prolonged Dex treatment, as its occupancy on the ChoRE of *Pklr* and *Fasn* genes was increased. Hepatic S1PR2 knockdown attenuated Dex-induced *Pklr* and *Fasn* gene expression as well as ChREBP recruitment to their ChoREs. Thus, S1PR2 mediated the chronic Dex-activated ChREBP to induce these two genes. As hepatic S1PR2 knockdown reduced the expression of ChREBPα and ChREBPβ, this could explain the decreased ChREBP occupancy on the ChoREs. Indeed, in mouse primary hepatocytes, short-term S1PR2 agonist treatment increased ChREBPα expression. This suggests that in chronic Dex treatment, S1PR2 signaling promotes ChREBPα expression initially, which eventually results in elevated ChREBPβ expression as ChREBPβ transcription can be activated by ChREBPα (28). It is important to note that hepatic S1PR2 knockdown only reduced a subset of Dex-induced ChREBP target genes (8 of 22 ChREBP target genes, Fig. 4*E*). It is unclear how this selectivity is achieved. Perhaps, chronic Dex treatment induces other pathways that activate ChREBPα and the increased ChREBPα expression that results from S1PR2 signaling can induce only certain ChREBPα target genes. In contrast to ChREBP target genes, hepatic S1PR2 knockdown suppressed most of the chronic Dex-induced Srebp1c target genes tested (Fig. 2*A*). Thus, S1PR2 signaling appears to play a more significant role in mediating chronic Dex treatment-activated Srebp1c than ChREBP action. More extensive analysis is needed to confirm this concept.

Interestingly, reducing ChREBP expression in mouse liver did not affect long-term Dex treatment-induced TG accumulation in the liver and the plasma, whereas Dex-induced lactate levels were attenuated. Thus, while ChREBP plays a major role in chronic Dex-induced glycolysis, its contribution to TG accumulation is modest. Gene expression analysis showed that hepatic ChREBP knockdown resulted in decreased expression of lipogenic genes, such as *Fasn, Me1*, and *Thrsp*, whereas some genes such as *Mttp* and *Glut2* were unaffected. However, this reduction was not sufficient to compromise the chronic Dex effect on lipid metabolism. Notably, *Srebp1c* expression in Dex-treated hepatic ChREBP knockdown mice is trending to be increased. This suggests a potential compensatory mechanism between the two transcription factors and can explain the inability of ChREBP knockdown alone to prevent chronic Dex-induced lipid disorders. Perhaps the increased *Srebp1c* expression limited the degree of reduction of lipogenic gene expression in the ChREBP knockdown liver. Thus, although lipogenic gene expression is decreased, the levels of reduction do not affect the rate of lipogenesis. Thus, there is a potential crosstalk between Srebp1c and ChREBP in mediating chronic Dex exposure-regulated lipogenesis. The interplay between Srebp1c and ChREBP in regulating lipogenic gene expression has been previously reported (32), though in this report hepatic *Srebp1c* expression was decreased in sucrose refed liver-specific ChREBP knockout mice (32). To further evaluate the potential interplay between Srebp1c and ChREBP in chronic Dex treatment-induced lipogenesis, future studies should investigate the effects of hepatic Srebp1c knockdown alone and in combination with ChREBP. Another possible explanation of these results is that other mechanisms such as the ceramide-PKCζ-Baf60c-USF1/2 axis might be sufficient to maintain chronic Dex response on hepatic lipid metabolism (16). A previous study found that liver-specific ChREBP knockout mice are protected from high-fat diet-induced hepatic steatosis (33). Our observation does not conflict with the previous studies, as the mechanisms by which a high-fat diet and prolonged glucocorticoid treatment lead to the development of hepatic steatosis are likely distinct.

Gene expression analysis showed chronic Dex treatment increased the expression of genes encoding enzymes in the ceramide and S1P biosynthetic pathways. These results were in agreement with our previous reports (12). Certain Dex-induced genes in the previous studies such as *Sptlc1/2* were not affected by Dex in these studies. This is likely due to the different Dex treatment regimens (4 mg/kg body weight for 2 weeks in this study *versus* 0.84 mg/kg body weight for 4 days in the previous study) (15). S1PR2 knockdown mostly antagonized the Dex effects and also reduced the basal expression of *Degs1* and *Sgpp1.* Notably, the impact of S1PR2 knockdown on the gene expression changes and its effect on ceramide and S1P metabolism requires further studies. It would be especially interesting to learn the effects of Dex treatment and S1PR2 knockdown on nuclear S1P levels, which have been previously shown to promote lipogenic gene transcription (22). Overall, these results suggest that there is another layer of mechanism by which S1PR2 depletion can suppress the lipogenic effect of chronic Dex exposure.

In summary, this study identified a unique mechanism of the role of S1PR2 signaling in exacerbating chronic glucocorticoid-induced lipid disorders by increasing the expression and activation of Srebp1c and ChREBPα. Elucidating signaling pathways that are solely activated under chronic glucocorticoid treatment is important for developing improved glucocorticoid therapy that has reduced adverse effects without affecting glucocorticoid anti-inflammatory activities. S1PR modulators have been investigated for the treatment of inflammatory diseases though there are no S1PR2 modulators in clinical use to date (34, 35). As S1PR2 antagonists could attenuate both glucose and lipid disorders induced by chronic glucocorticoid exposure, a combinatorial approach of using glucocorticoids and S1PR2 antagonists could potentially improve current glucocorticoid therapy.

## Experimental procedures

### Animals

All mice used for this study were male and 8 to 12 weeks old of mixed C57Bl6/J:129SvJ background. Mice were bred and cohoused in groups of 2 to 5 mice per cage. Each cage contains Sanichip bedding, cotton Nestlet, and crinkled paper. Mice had free access to water and standard chow diet (5053 PicoLab, Rodent Diet 20, LabDiet) and were housed in a 13 h/11 h light/dark cycle. Mice were fasted with free access to water at ZT2 and blood was collected at ZT8. Tissue collection occurred *ad libitum* between ZT4-ZT6. Tissues were snap frozen in liquid nitrogen and stored at −80 °C until analysis. Fat and lean mass composition were measured using Echo MRI Whole Body Composition Analyzer. All experiments performed are approved by the Animal Care and Use Committee at University of California, Berkeley (AUP-2014-08-66173).

### Adeno-associated virus

Adeno-associated virus serotype 8 (AAV8) expressing control scramble shRNA or shRNA targeting *S1pr2* and *Chrebp* were purchased from VectorBuilder. shRNA sequences are as follows: *S1pr2* under U6 promoter GCCATCGTGGTGGAGAATCTT, *Chrebp* under miR30 promoter CACCCACATCTTCAAACTACAT. Mice were injected through the tail vein with 3 x 10^11^ GC for U6 promoter and 4 x 10^11^ GC for miR30 promoter.

### Dexamethasone administration

Ten days after AAV8 injection, mice were treated with 4 mg/kg body weight of Dexamethasone for 14 days through drinking water. Dexamethasone sodium phosphate (PHR1768, Sigma-Aldrich) was dissolved in drinking water as previously described (15).

### Primary hepatocyte isolation

Immediately after euthanasia, the inferior vena cava was cannulated and the portal vein was cut. The liver was first perfused with 40 ml HBSS without calcium and magnesium, supplemented with 0.5 mM EDTA at a flow rate of 5 ml/minute with periodic clamping of the portal vein. Liver was then perfused with 25 ml of Type I Collagenase (17100017, Gibco) at a concentration of 0.3 mg/ml in phenol red-free and pyruvate-free DMEM. Solutions were maintained at 37 °C. Liver was excised and collected in 10 ml of DMEM with 0.3 mg/ml Type I Collagenase. Liver lobes were carefully torn to release hepatocytes. Media is strained through 70 μM and 45 μM cell strainers to isolate cells before undergoing centrifugation to isolate hepatocytes as previously reported (36). Hepatocytes were plated in 10% FBS supplemented DMEM on collagen-coated plates and incubated at 5% CO_2_ and 37 °C. Hepatocytes were treated with DMSO, 500nM Dexamethasone (D4902, Sigma Aldrich), and 1mM of CYM-5520 (HY-100953, MedChemExpress) for 4 hours before RNA isolation.

### Triglyceride quantification

Blood was collected in EDTA-coated microvettes (16.44.100; Sarstedt) and spun at 2000g for 15 min at 4 °C to isolate plasma. 50 mg of liver was donce homogenized, boiled at 100 °C for 5 min, placed on ice for 2 min, boiled for an additional 5 min, and spun at 10,000g at room temperature for 10 min. Supernatant was transferred to new tubes. Triglycerides were measured using a triglyceride colorimetric assay kit (10010303; Cayman Chemicals) according to manufacturer’s instructions.

### Histology staining

Livers were fixed in 4% paraformaldehyde for 24 h, dehydrated in 70% ethanol for 24 h before hematoxylin and eosin staining by the Liver Tissue Analysis Core at the UCSF Liver Center. For Oil Red O staining, livers were frozen in OCT, cryosectioned, and stained by the Gladstone Institutes Histology and Light Microscopy Core.

### Western blot

Tissue samples were homogenized using BeadBug with four cycles of 30 s at 2500 speed in RIPA supplemented with protease and phosphatase inhibitors (4693159001, 4906845001; Sigma Aldrich). Homogenate was centrifuged at 17,000*g* for 15 min at 4 °C. Supernatant was transferred and protein concentration was quantified using Pierce BCA Protein Assay Kit (23228; ThermoFisher). 30 μg protein was mixed with 4x Laemmli SDS-sample Buffer (Boston Bioproducts, Milford, PA) and 10x NuPage Sample Reducing Agent (NP0004; ThermoFisher), ran on handcasted 10% SDS polyacrylamide gels, and blotted on nitrocellulose membrane. Membranes were blocked in 10% dry milk and incubated at 4 °C overnight with primary antibody (1:1000). The next day membranes were washed with TBST (0.1% Tween-20) and incubated with Licor IRDye 800CW Goat anti-rabbit Secondary Antibody (1:10,000) for 1 h before visualization using LiCor Odyssey Imager. Band density was quantified using ImageJ software. Primary antibodies used are as follows: Alpha Tubulin (11224-1-AP, Proteintech, Rosemont, IL), S1PR2 (21180-1-AP, Proteintech), SPHK2 (ABS526, Millipore Sigma), ChREBP (NB400-135, Novus Biologicals).

### Chromatin immunoprecipitation

All buffers were supplemented with protease inhibitors (535140, Millipore Sigma) upon use. Buffer contents are as previously reported (15). 300 mg liver was minced in 10 ml 1x SSC buffer and pelleted at 1700g at 4 °C for 3 min and crosslinked with 1% formaldehyde for 10 min at room temperature with gentle agitation before quenched with 125 mM glycine, pelleted, and washed twice with PBS. The pellet was resuspended in 5 ml hypotonic buffer (10 mM HEPES pH 7.9, 15 mM MgCl_2_, 10 mM KCl, 0.2% NP-40, 1 mM EDTA, 5% sucrose) supplemented with spermine and spermidine for 5 min and homogenized with 10 strikes with a glass dounce homogenizer. Lysate was mounted on 5 ml cushion buffer (10 mM Tris pH7.5, 15 mM NaCl, 60 mM KCl, 1 mM EDTA, 10% sucrose), supplemented with spermine and spermidine, and pelleted at 1700g at 4 °C for 8 min to isolate nuclei. Nuclei was resuspended in 3 ml sonication buffer (50 mM Tris ph8, 2 mM EDTA, 0.1% Triton X-100) and sonicated at 45% amplitude for 10 s bursts with 60 s rests. The total sonication on time was 5 min. Sonicated product was centrifuged at 17,000g for 15 min at 4 °C to remove debris. Supernatant was diluted with one volume of dilution buffer (20 mM Tris pH8, 2 mM EDTA, 200 mM NaCl, 0.1% Na-deoxycholate) and divided for IP. Antibodies were incubated overnight at 4 °C with rotation at a concentration of 4 μg. Antibodies were: IgG (A01008,GenScript), rabbit polyclonal antibody against GR (N499), SREBP-1 (sc-13551, Santa Cruz Biotechnology), and ChREBP (NB400–135, Novus Biologicals). 40 μl of A/G beads (sc-2003, Santa Cruz Biotechnology) were incubated for 2 h with rotation at 4 °C. Beads were washed with the following buffers for 5 min, rotating: 500 μl of Tris-Saline-EDTA (TSE) Buffer I (0.5% Triton X-100, 2 mM EDTA, 20 mM Tris pH8, 150 mM NaCl), TSE II (0.5% Triton X-100, 2 mM EDTA, 20 mM Tris pH8, 500 mM NaCl), TSE III (0.25 M LiCl, 1% NP-40, 1% Na-deoxycholate, 1 mM EDTA, 10 mM Tris pH8) and Tris-EDTA (TE) buffer (10 mM Tris ph8, 1 mM EDTA) and centrifugation at 3000*g* for 1 min between washes. IPs were resuspended in 400 μl of elution buffer (100 mM NaHCO_3_, 1% SDS) and rotated for 1 h at room temperature. A final concentration of 200 mM NaCl was added to each sample before incubating in 65 °C overnight. Samples were then incubated at 55^o^C for 1 h with 8 μl 0.5 M EDTA, 16 μl 1M Tris HCl pH 6.5, 1.5 μl Proteinase K (E00491, Thermo Fisher, Waltham MA) and 1 μl RNase A (EN0531, Thermo Fisher, Waltham, MA) before PCR cleanup. DNA was eluted with 35 μl autoclaved water. ChIP RT-PCR primers are listed in Table 1.

**Table 1:** ChIP RT-PCR primers (mouse)

Gene	Forward	Reverse
*Fasn* SRE	TTTAAAGGGAGGGAGGGAGA	GGCACTGATAGGGAAACACTGA
*Pklr* ChoRE	AGTCTGCTTTAAGTGGGGCTC	GATCCCCTTTGGCCTGTCTG
*Fasn* ChoRE	GGCCTGCTCCACATCGAAA	GGCCCATCACCCTATTGCCT
*Mttp* ChoRE	GGGCAAAGAATCTACCACCA	TTCCAGAGGCTTTCAAGGAG
*Rpl19*	TCCTTGGTCTTAGACCTGCG	ATGGAGCACATCCACAAGC

### Real-time PCR

Total RNA was isolated using TriReagent (T0424, Sigma Aldrich). Reverse transcription was done as follows: Mixing a total volume of 16 μl of: 1000 ng of total RNA, 4 μl of 2.5 mM dnTP and 2 μl of 15 μM random primers (S1254S, New England Biolabs) and incubating at 70 °C for 5 min. Then a cocktail containing 25 units of M-MuLV Reverse Transcriptase, 10 units of RiboLock and 2 μl of 10x M-MuLV Reverse Transcriptase Reaction Buffer were added and samples were incubated at 42 °C for 1 h. The cDNA was diluted 1:5 and used for quantitative PCR using Power EVA qPCR Supermix Kit (K5057400, Biochain). Real-time PCR was performed on the CFX Opus 96 (BioRad). mRNA levels were normalized to rpl19. Primers are listed in Table 2.

**Table 2:** RT-PCR primers (mouse)

Gene	Forward	Reverse
Rpl19	TCCTTGGTCTTAGACCTGCG	ATGGAGCACATCCACAAGC
Fasn	GGCATCATTGGGCACTCCTT	GCTGCAAGCACAGCCTCTCT
ChREBPα	CGACACTCACCCACCTCTTC	TTGTTCAGCCGGATCTTGTC
ChREBPβ	TCTGCAGATCGCGTGGAG	CCTGTCCCGGCATAGCAAC
Pklr	AGATGCAACATGCGATTGCC	GCACAGCACTTGAAGGAAGC
Mttp	CTCTTGGCAGTGCTTTTTCTCT	GAGCTTGTATAGCCGCTCATT
Elovl6	GAAAAGCAGTTCAACGAGAACG	AGATGCCGACCACCAAAGATA
Srebp1c	GGAGCCATGGATTGCACATT	GGCCCGGGAAGTCACTGT
Dgat2	AGTGGCAATGCTATCATCGT	AAGGAATAAGTGGGGAACCCAGATCA
Gpat1	GCTATCATGTCCACCCACATTG	ACTTCCTCCTTCATCACAAAGAAGTC
Cd36	TTGAAAAGTCTCGGACATTGAG	TCAGATCCGAACACAGCGTA
Acaca	CCTCCGTCAGCTCAGATACA	TTTACTAGGTGCAAGCCAGACA
Acacb	CGCTCACCAACAGTAAGGTGG	GCTTGGCAGGGAGTTCCTC
Me1	GTCGTGCATCTCTCACAGAAG	TGAGGGCAGTTGGTTTTATCTTT
Thrsp	ATGCAAGTGCTAACGAAACGC	CCTGCCATTCCTCCCTTGG
Scd1	TTCTTGCGATACACTCTGGTGC	CGGGATTGAATGTTCTTGTCGT
Lipg	ATGCGAAACACGGTTTTCCTG	GTAGCTGGTACTCCAGTGGG
Glut2	TTCCAGTTCGGCTATGACATCG	CTGGTGTGACTGTAAGTGGGG
G6pc1	ATGACTTTGGGATCCAGTCG	TGGAACCAGATGGGAAAGAG

### Lactate assay

Lactate levels were measured in 5 to 10 mg pieces of homogenized liver using the Lactate-Glo Assay according to manufacturer’s instructions (J5021, Promega, Madison, WI).

### RNA-sequencing

Three biological replicates per treatment group were used. Liver total RNA was extracted using a Direct-zol Microprep Kit (Zymo Research). Samples were sent to Innomics (Sunnyvale, CA) for sequencing. Briefly, library preparation was performed using the Optimal Dual-mode mRNA Library Prep kit (BGI-Shenzhen, China). 200 ng of RNA was denatured and mRNA was enriched by oligo-attached magnetic beads before fragmentation and cDNA synthesis. Library adaptors were ligated to the ends of the cDNA and amplified through PCR before quality control. Products were sequenced with PE150 base length reads on DNBSEQ-T7. The final clean data undergoes quality control by SOPAnuke and the final clean data amount has about 20 million read pars per sample. The reference genome used was GCF_000001635.27_GRCm39. A full list of RNA-seq-identified significant differentially expressed genes without fold cut-off are listed in File S1. The RNA-seq data has been deposited in the NCBI Gene Expression Omnibus Database under accession code, GSE286363. Gene ontology analysis was categorized using NIH’s DAVID Bioinformatics program.

### Statistics

Data was analyzed using Prism 10 software (Graphpad). Individual data points represent independent biological replicates. Error bars represent standard deviation (SD). Bar graphs represent mean. Statistical comparisons used are stated in figure legends, using unpaired *t* test with Welch’s correction or two-way ANOVA with Fisher’s LSD tests.

## Data availability

RNA-seq data is deposited in GEO with accession number GSE286363. Data is available upon request.

## Supporting information

This article contains supporting information.

## Conflict of interest

The authors declare that they have no conflicts of interest with the contents of this article.
